# Supplementary material for: Discovery and characterization of a pigment produced by Streptococcus pyogenes
Source: mBio. 2026 Sep 23;17(10):e01348-26. doi: 10.1128/mbio.01348-26 (PMC13712749; doi:10.1128/mbio.01348-26)
Supplement: Supplementary file 1 [file mbio.01348-26-s0001.pdf]

SUPPLEMENTAL INFORMATION

Discovery and characterization of a pigment produced by *Streptococcus pyogenes*

Sobita Pathak, Theo DeVinney, Artemis Gogos, Reginald A. Woods, Caleb M. Anderson, Jennifer C. Chang, Michael J. Federle

University of Illinois at Chicago, Chicago, Illinois

TOC

Supplemental Figures : **Page**

Figure S1. Absorbance in culture supernatants and cell extracts of pigmented and non-pigmented strains 2

Figure S2. Heat stability of the pigment in cell pellets. 3

Figure S3. UV-visible light absorbance of MGAS5005 and *pfl*::IS*S1* mutant strains 3

Figure S4. Pigment phenotypes of *pfl*, *nupPQ*, and *yigB*. 4

Figure S5. UV-Vis absorption spectra and HPLC chromatograms of riboflavin and GAS pigment. 5

Figure S6. Supernatant complementation between non-pigmented *S. pyogenes* mutants. 6

Figure S7. Role of metals in pigment biosynthesis. 7

Figure S8. Effect of complex media and media components on pigment production. 8

Figure S9. Size exclusion chromatography of MGAS5005 pigment extract. 8

Figure S10. Nitric oxide response to pigment extracts 9

Figure S11. Total antioxidant capacity of pigmented and non-pigmented extracts. 10

Figure S12. Competitive fitness of pigmented and non-pigmented strains under aerobic and anaerobic growth conditions. 10

Figure S13. Antibacterial test of pigmented and non-pigmented extracts 11

Figure S14. Hemolytic activity of pigmented and non-pigmented strains 12

Table S1. Strains used in this study 13

Table S2. Plasmids and Primers used in this study 14

Table S3. Strain library assessed for pigment production 15

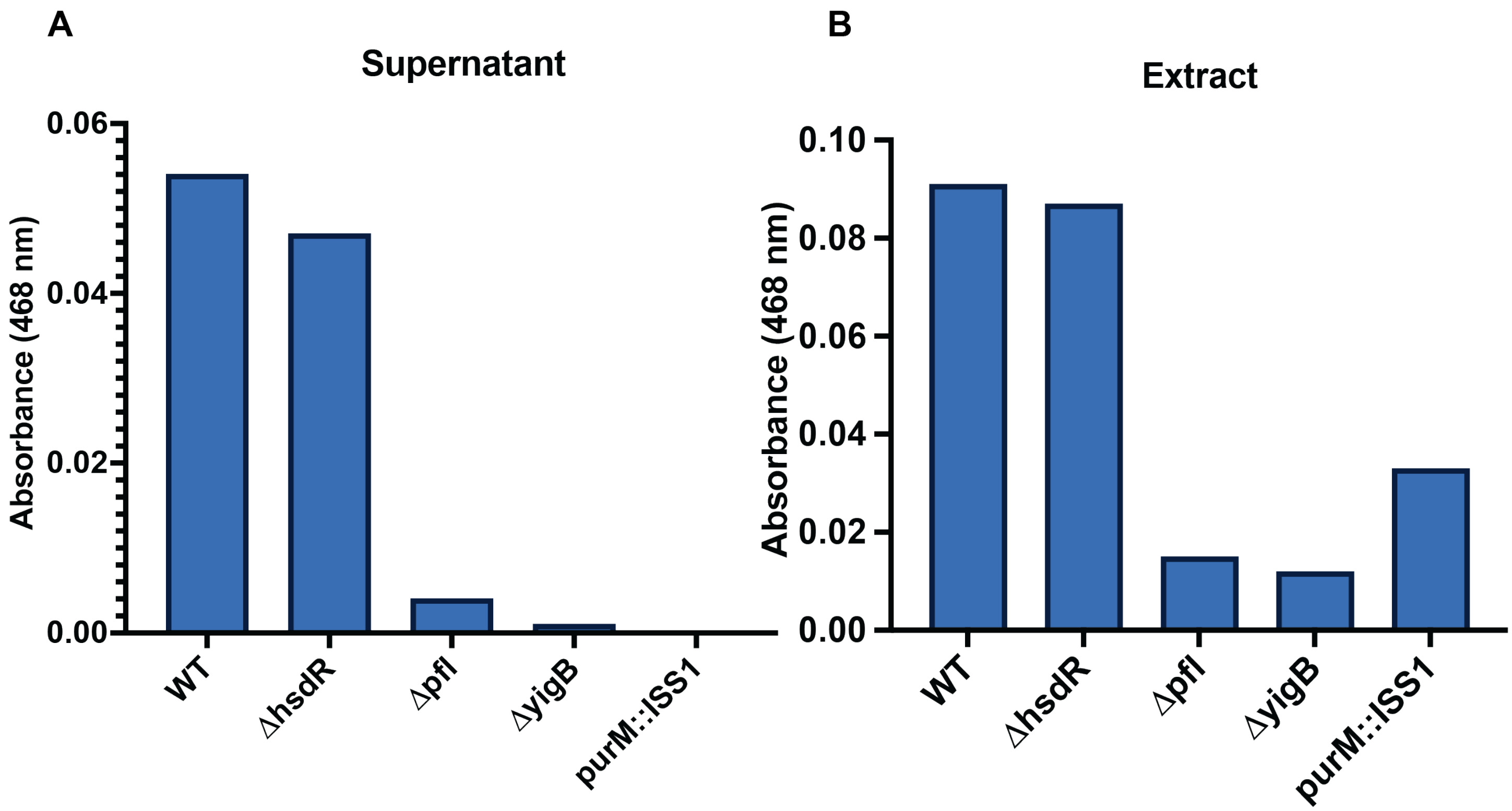

Figure S1. Absorbance in culture supernatants and cell extracts of pigmented and non-pigmented strains. Absorbance at 468 nm was measured in the culture supernatant (left) and cell-associated extract (right) of WT, Δ*hsdR*, Δ*pfl*, Δ*yigB*, and *purM*::ISS1 strains following growth in CDM. Pigmented strain (WT and Δ*hsdR*) exhibited the highest absorbance in both fractions, whereas substantially lower absorbance was observed for the non-pigmented mutants. Low but detectable absorbance was present in the supernatants of WT and Δ*hsdR*, while the corresponding cell extracts showed higher absorbance, consistent with the pigment being predominantly cell-associated with a smaller amount present in the culture supernatant.

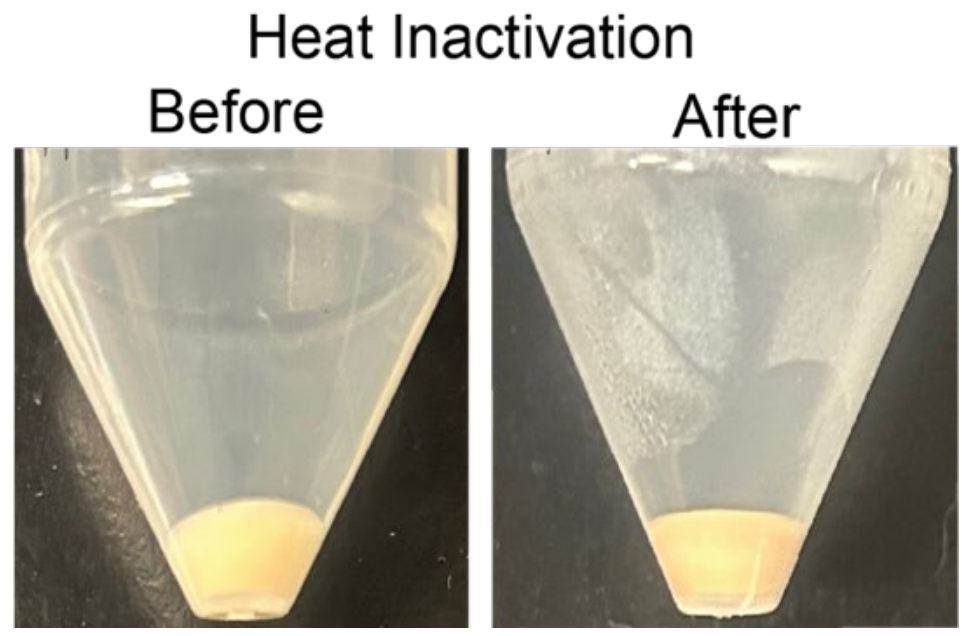

Figure S2. Heat stability of the pigment in cell pellets. The pellet on the left shows the fully developed pigment prior to heat treatment, while the pellet on the right retained similar coloration after heat inactivation at 80 °C for 10 min.

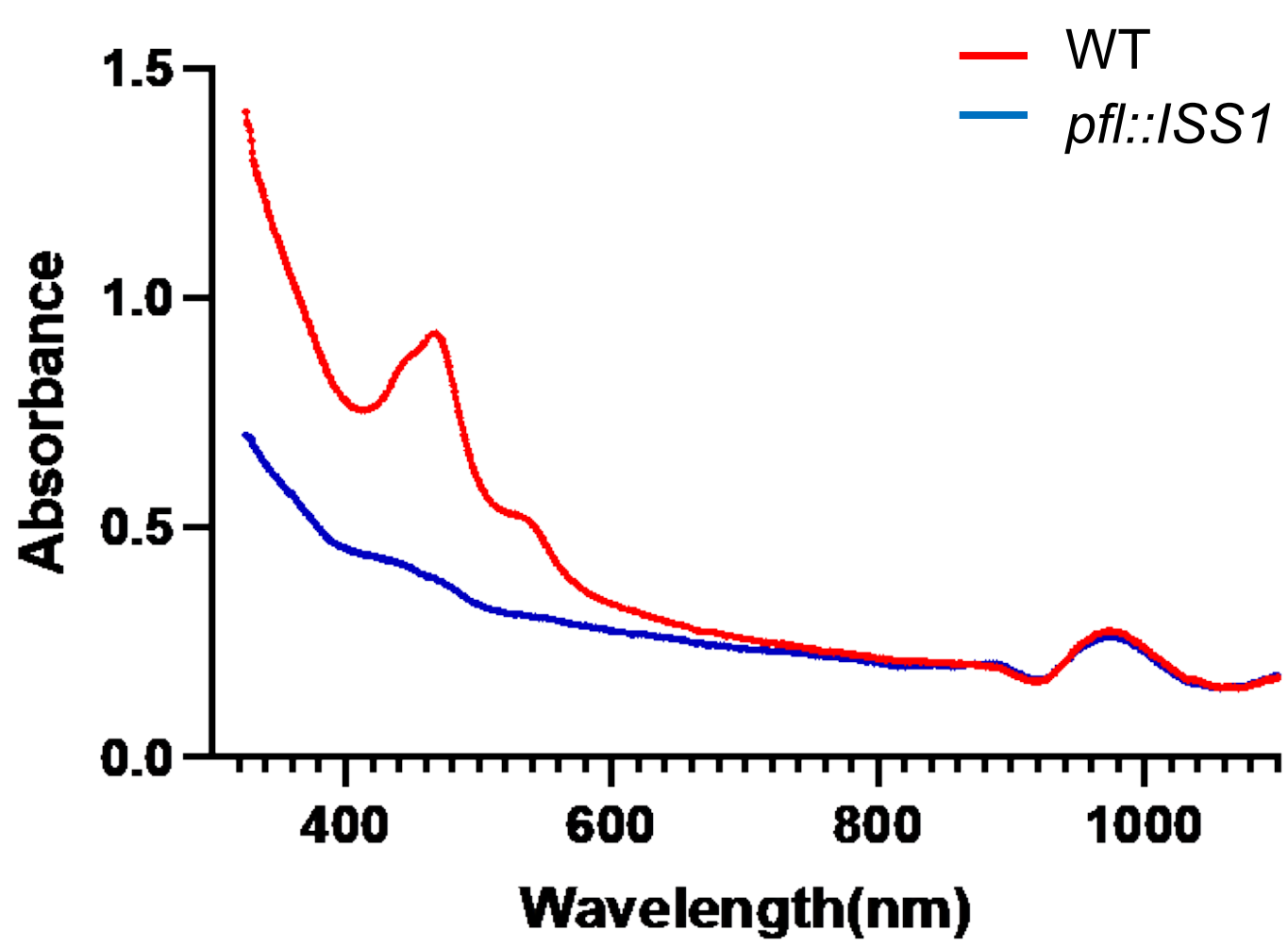

Figure S3. UV-visible light absorbance comparison between MGAS5005 and *pfl*::IS*S1* mutant strains. UV – Vis absorption spectra of filtered extracts (1 mL) were recorded over a wavelength range of 300 - 1100 nm, using MeOH + 0.1% Formic Acid as the blank. WT (MGAS5005) extract exhibited a characteristic absorption peak at 468 nm whereas no corresponding peak was observed in the *pfl::ISS1* mutant.

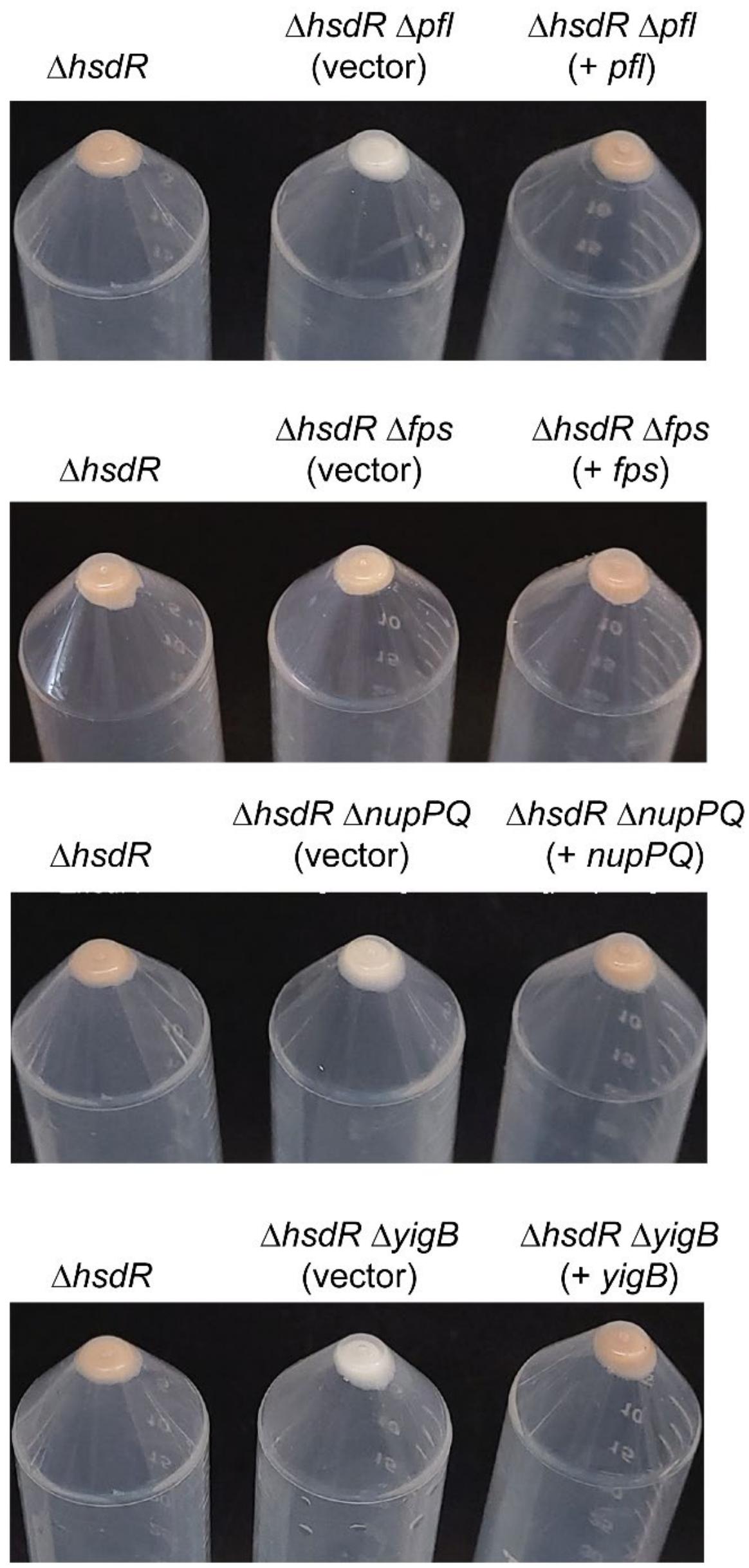

Figure S4. Pigment phenotypes of *pfl*, *fps*, *nupPQ*, and *yigB*. In-frame deletions were generated in the MGAS5005 hsdR background. Complementation of each gene was provided on plasmid pLZ12-spec, which served as the vector control.

A

B

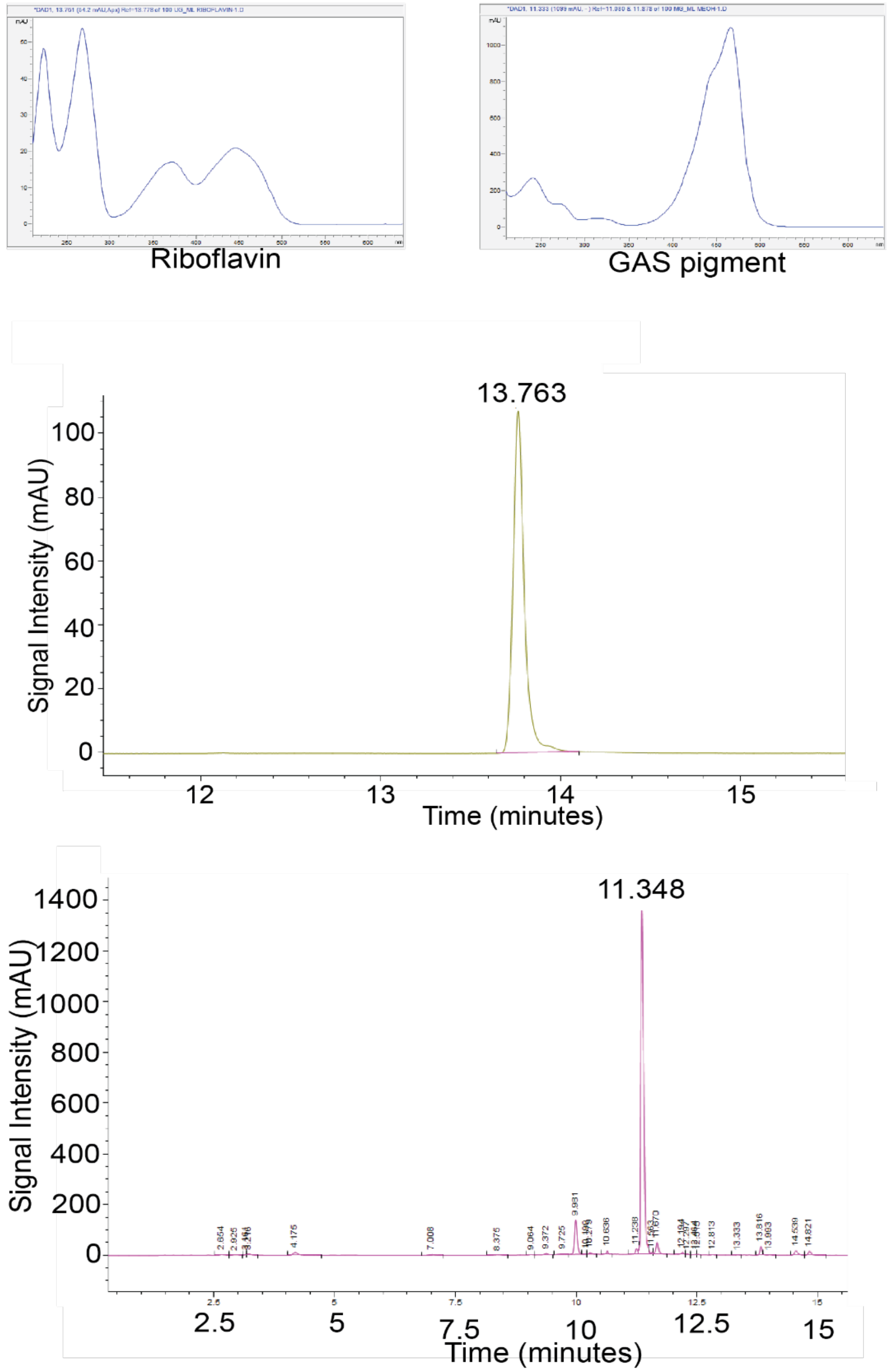

Figure S5. UV-Vis absorption spectra and HPLC chromatograms of riboflavin and GAS pigment. A) UV–Vis absorption spectra of riboflavin (left) and the GAS pigment extract (right). Riboflavin displayed characteristic absorption peaks in the UV and visible regions at 223, 266, 373, and 445 nm, whereas the GAS pigment has a distinct absorption maximum at 468 nm. B) HPLC chromatograms monitored at 468 nm for riboflavin (top chromatogram) and the GAS pigment extract (bottom chromatogram). Riboflavin eluted at a retention time of 13.763 min, while the GAS pigment eluted at 11.348 min. The differences in both UV–Vis spectral profile and HPLC retention time indicate that the GAS pigment is distinct from riboflavin.

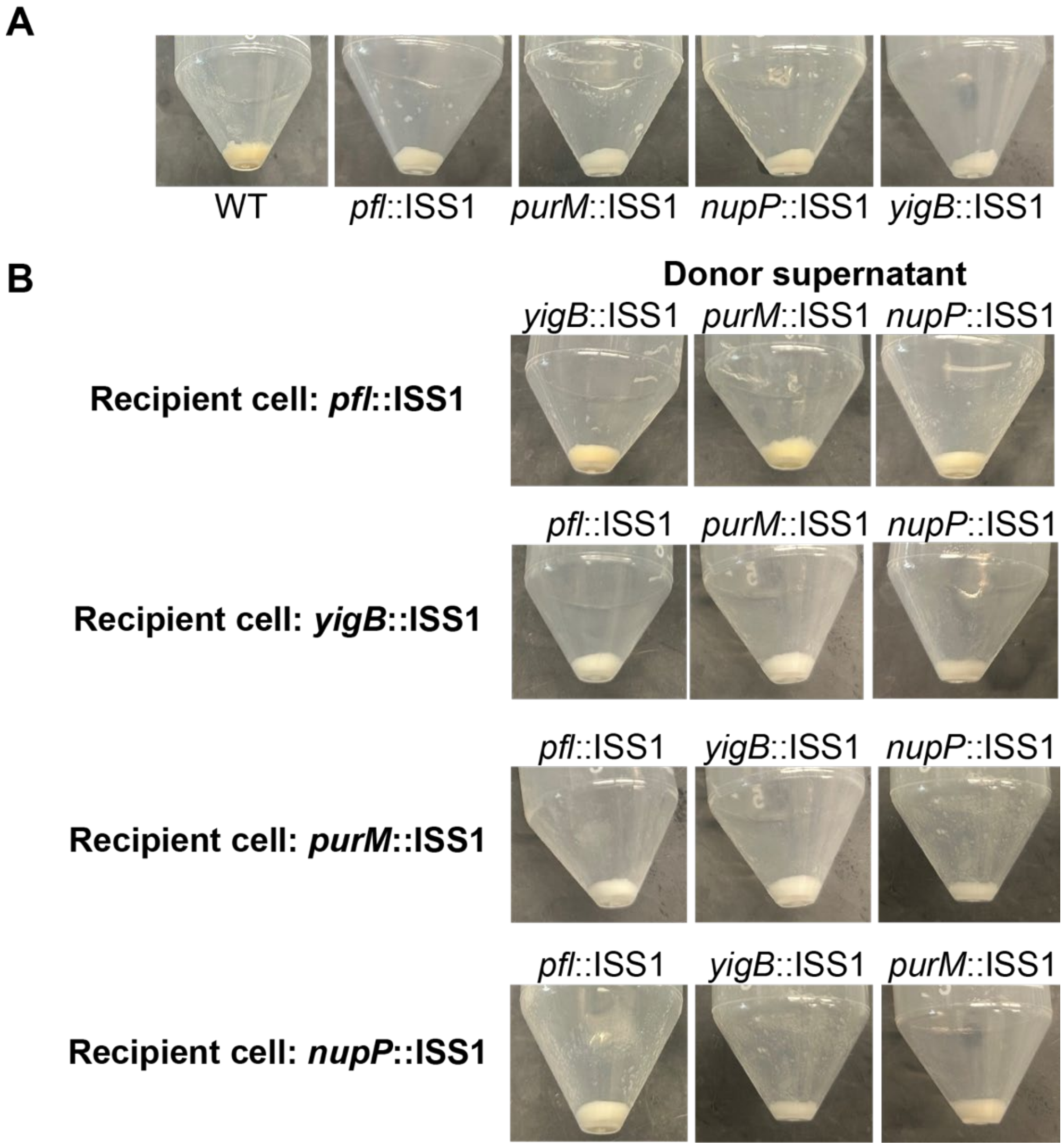

Figure S6. Supernatant complementation between non-pigmented *S. pyogenes* mutants. (A) Individual strains cultured in CDM and pelleted. Culture supernatants were used as spent-media donor material. (B) Pellet pigmentation following growth of recipient mutant strains in CDM supplemented with 50% cell-free culture supernatant obtained from the indicated donor mutants. Recipient strains and donor supernatants are indicated for each condition. Changes in pellet coloration were assessed to determine whether extracellular components produced by one non-pigmented mutant could restore pigmentation in another non-pigmented mutant background.

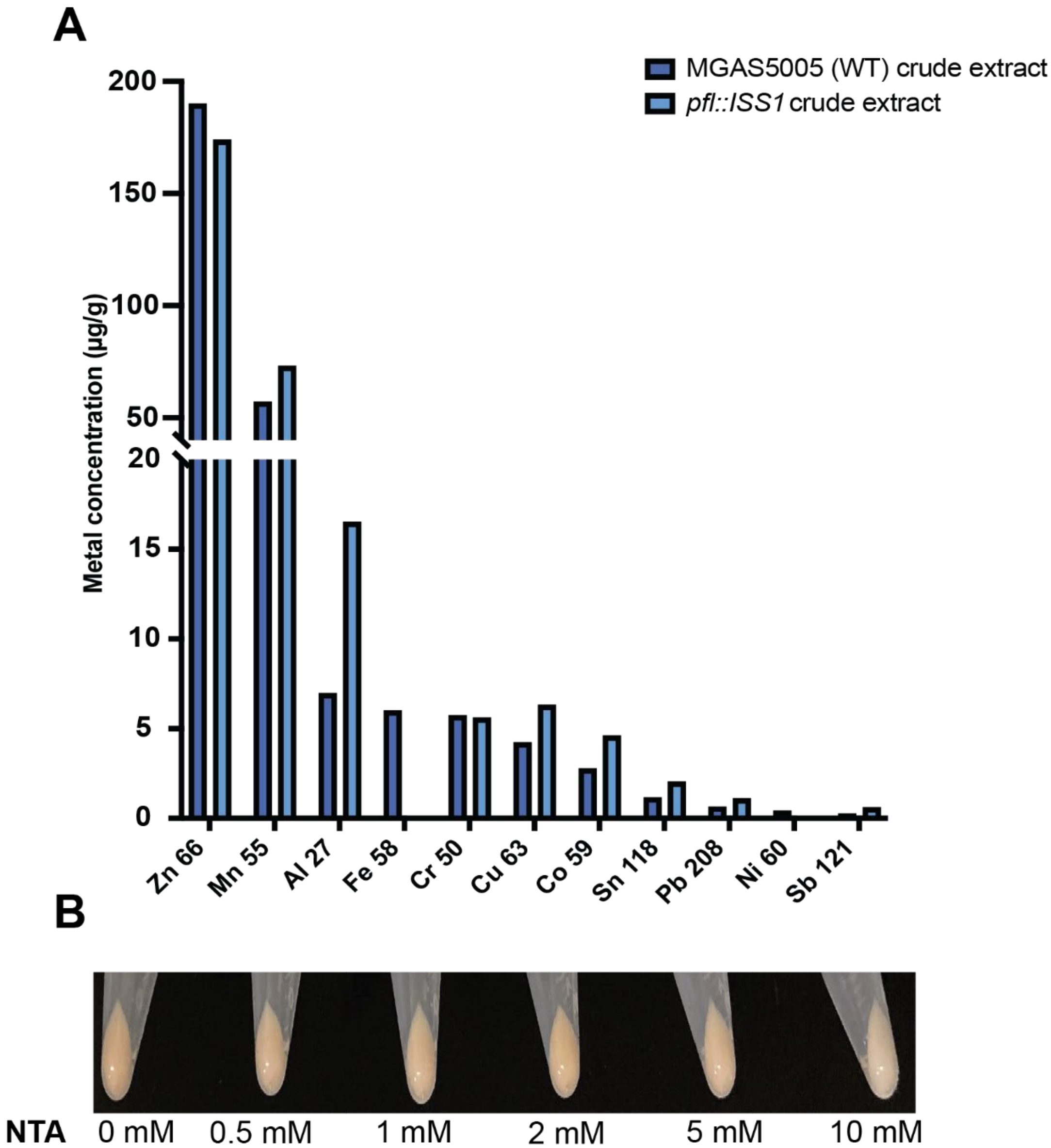

Figure S7. Role of metals in pigment biosynthesis. (A) Metal concentrations in crude extracts isolated from *S. pyogenes* MGAS5005 (WT) and *pfl*::ISS1 cultures were measured by elemental analysis. Concentrations of Zn, Mn, Al, Fe, Cr, Cu, Co, Sn, Pb, Ni, and Sb are shown in µg/g. The y-axis is discontinuous to facilitate visualization of metals present at lower concentrations. Representative images of the corresponding crude extracts are shown below the graph. ICP-MS comparison between pigmented and non-pigmented crude extract revealed a significant difference in the levels of iron (Fe) examined between the two samples. (B) NTA addition (to final concentration indicated) reduced pigmentation in a concentration-dependent manner.

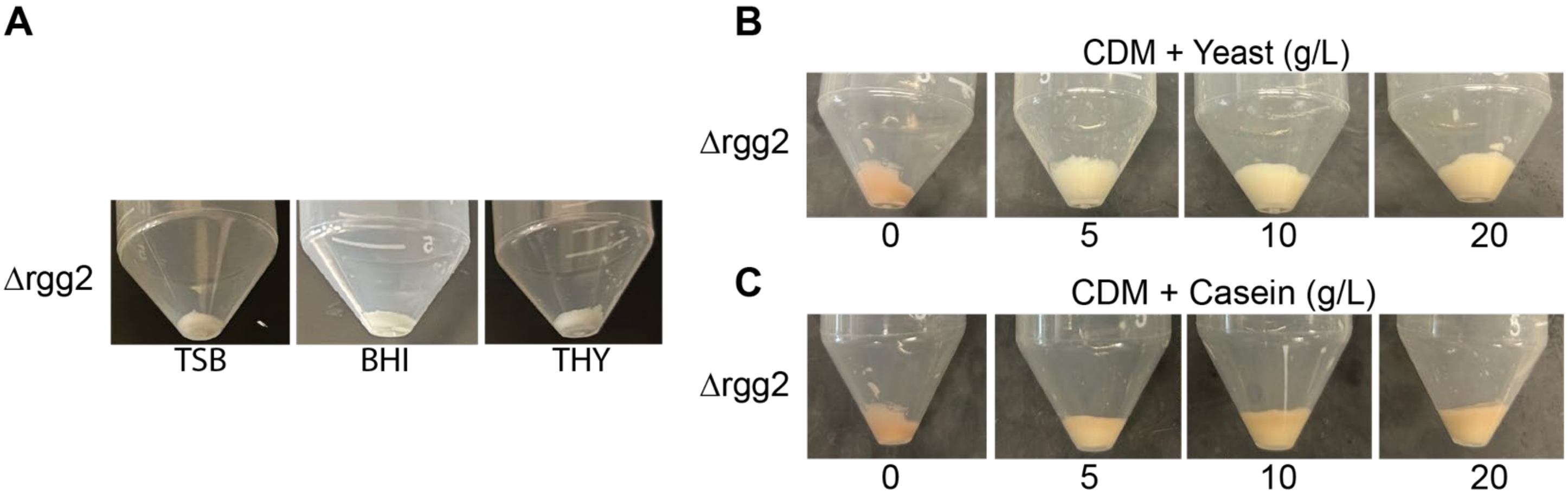

Figure S8. Effect of complex media and media components on pigment production. (A) Pigmentation was assessed following growth in the complex media Tryptic Soy Broth (TSB) and Brain Heart Infusion (BHI), in which no visible pigmentation was observed. To further investigate the effect of rich-media components, CDM was supplemented with increasing concentrations of yeast extract and casein hydrolysate. Increasing concentrations of yeast extract resulted in complete suppression of pigmentation, whereas casein hydrolysate only partially reduced pigmentation.

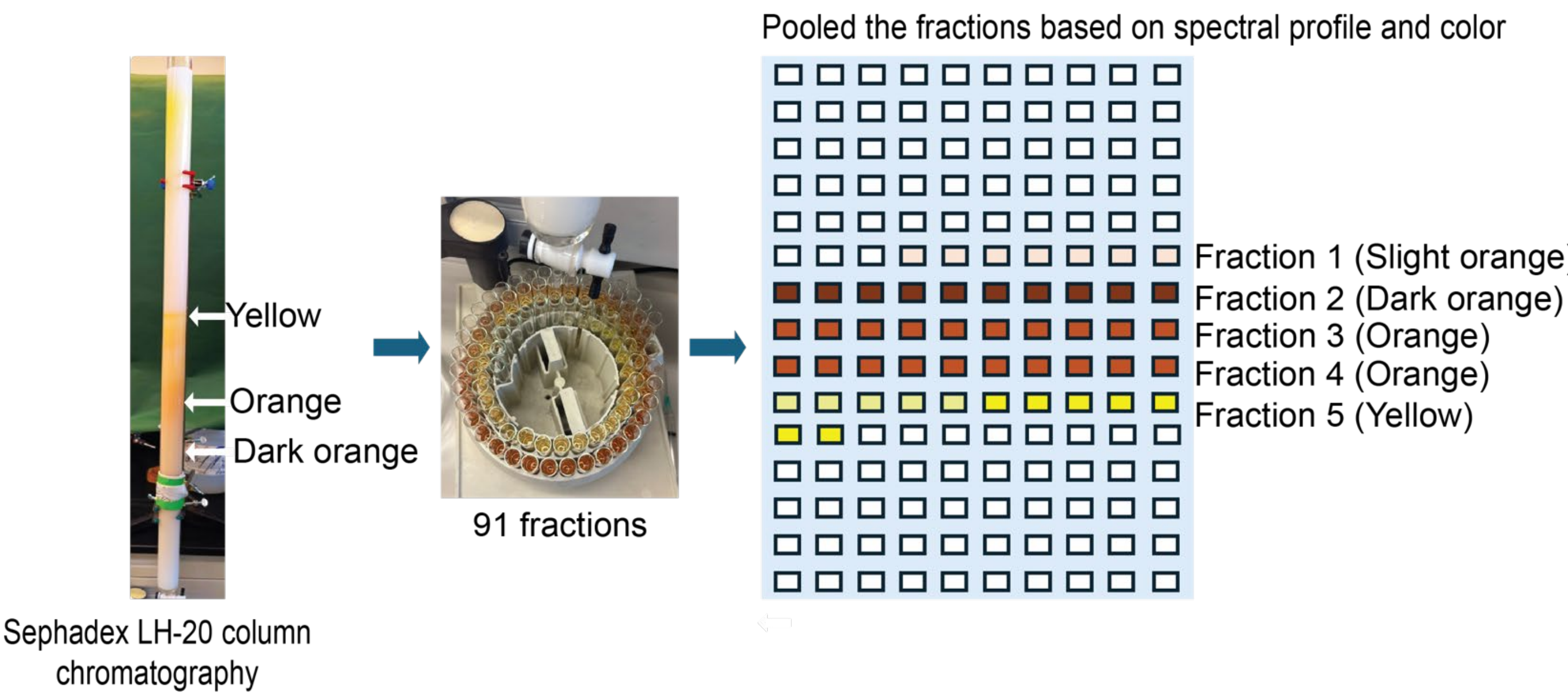

Figure S9. Size exclusion chromatography of MGAS5005 pigment extract. A Sephadex LH-20 column (120 cm height) was used for chromatography. During elution, fractions displaying distinct color differences were observed along the column (left panel, arrows). Eluted fractions were collected into individual tubes (middle panel). A total of 91 fractions were obtained and subsequently pooled based on similarities in color and UV - Vis spectral profiles (right panel). The pooled fractions were categorized into major groups, including dark orange, orange, and yellow fractions.

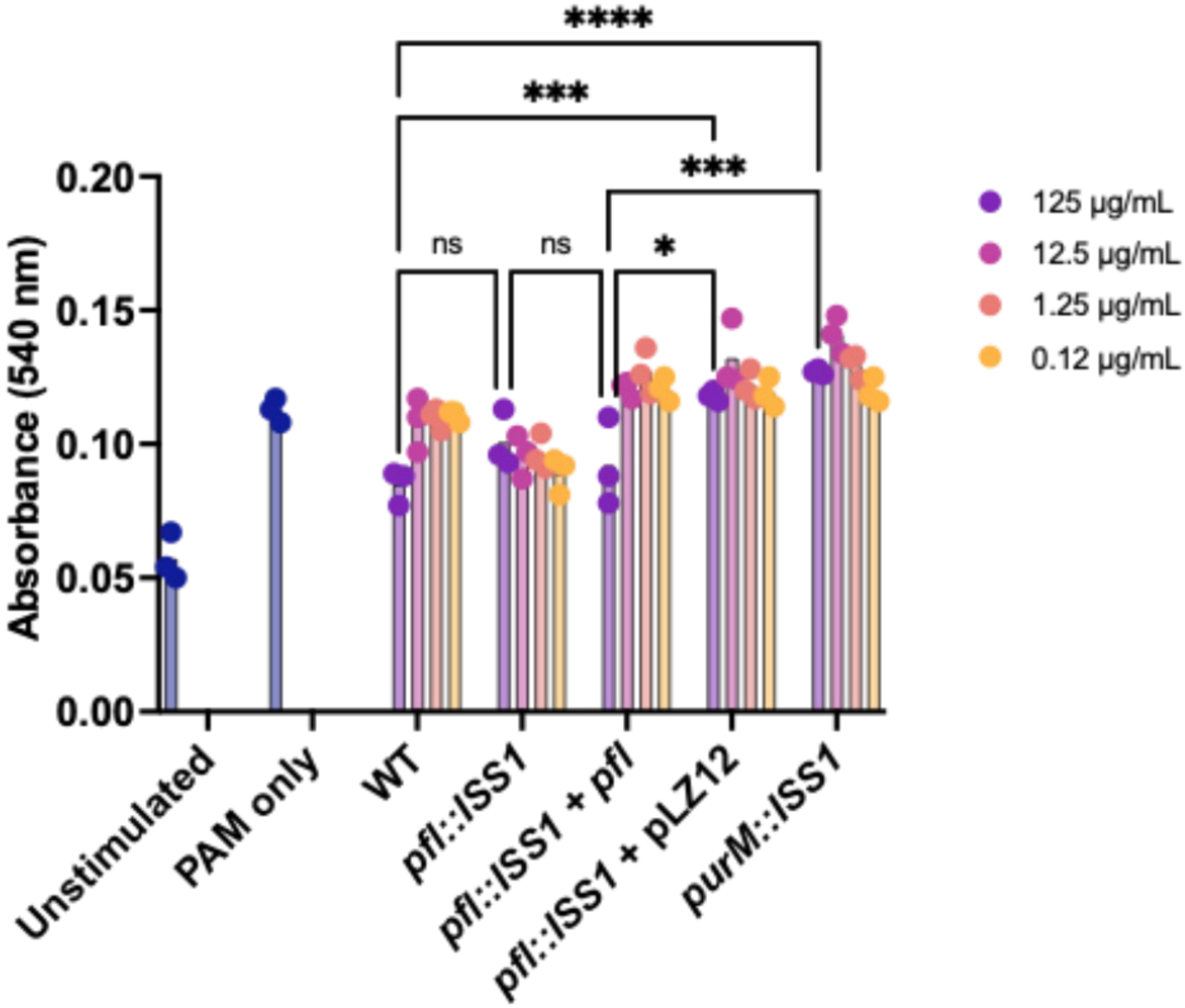

Figure S10. Nitric oxide response to pigment extracts. Nitrite levels in PAM3CSK4-treated cells were quantified following treatment with pigmented and non-pigmented extracts under different concentrations (500, 50, 5, 0.5 µg/mL). Data are shown as means ± SD from three independent experiments (n = 3). Statistical significance was determined by two-way ANOVA with Tukey's multiple-comparisons test. *, $p < 0.05$; **, $p < 0.01$; ***, $p < 0.001$; ****, $p < 0.0001$.

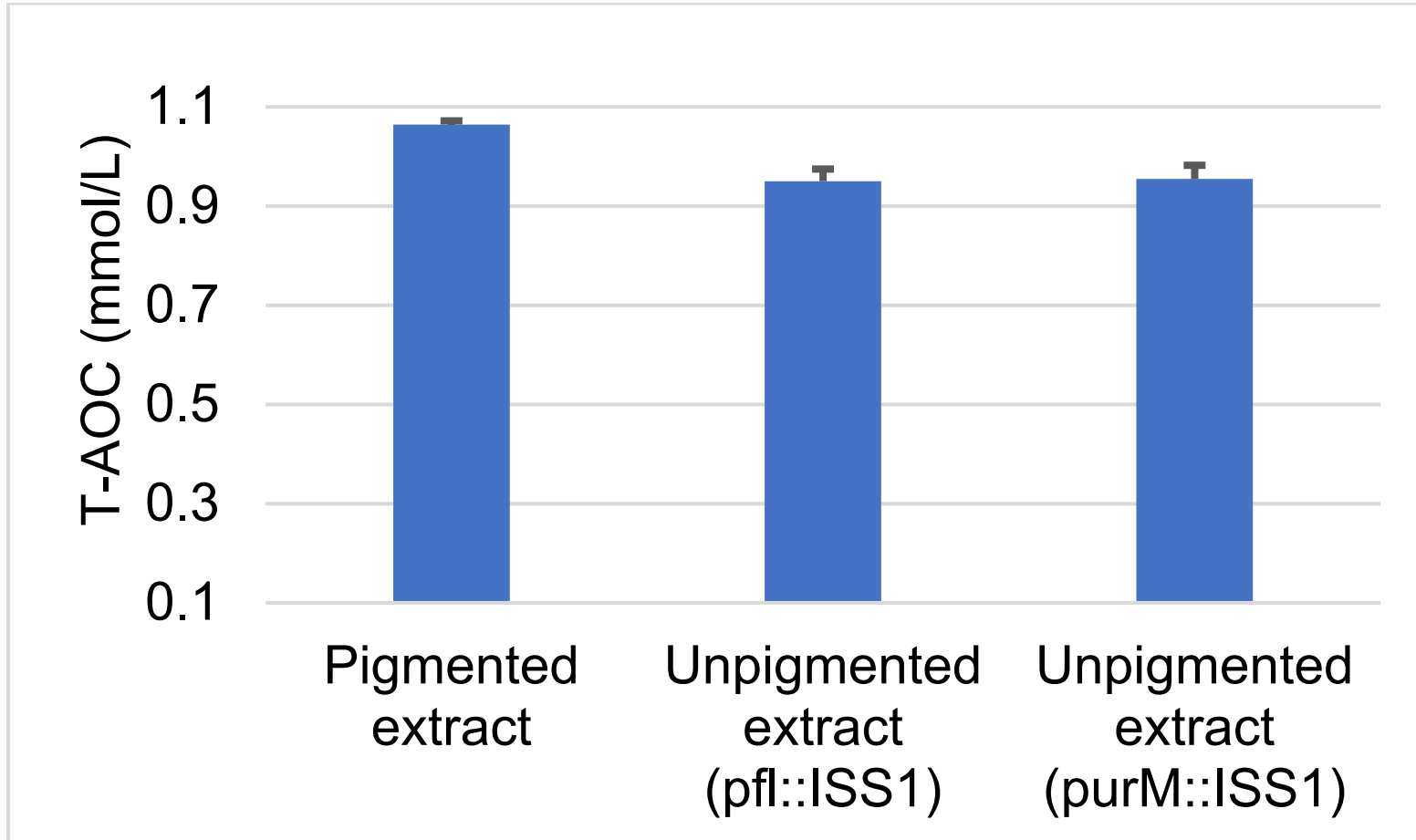

Figure S11. Total antioxidant capacity of pigmented and non-pigmented extracts. Total antioxidant capacity (TAC) was measured using commercial assay kit (Abcam). Pigment was extracted from WT (MGAS5005) strain while non-pigmented extracts were extracted from the *pfl::ISS1* (isoprenoid pathway mutant) and *purM::ISS1* (purine biosynthesis mutant) strains. No significant difference was observed between pigmented and both non-pigmented extracts.

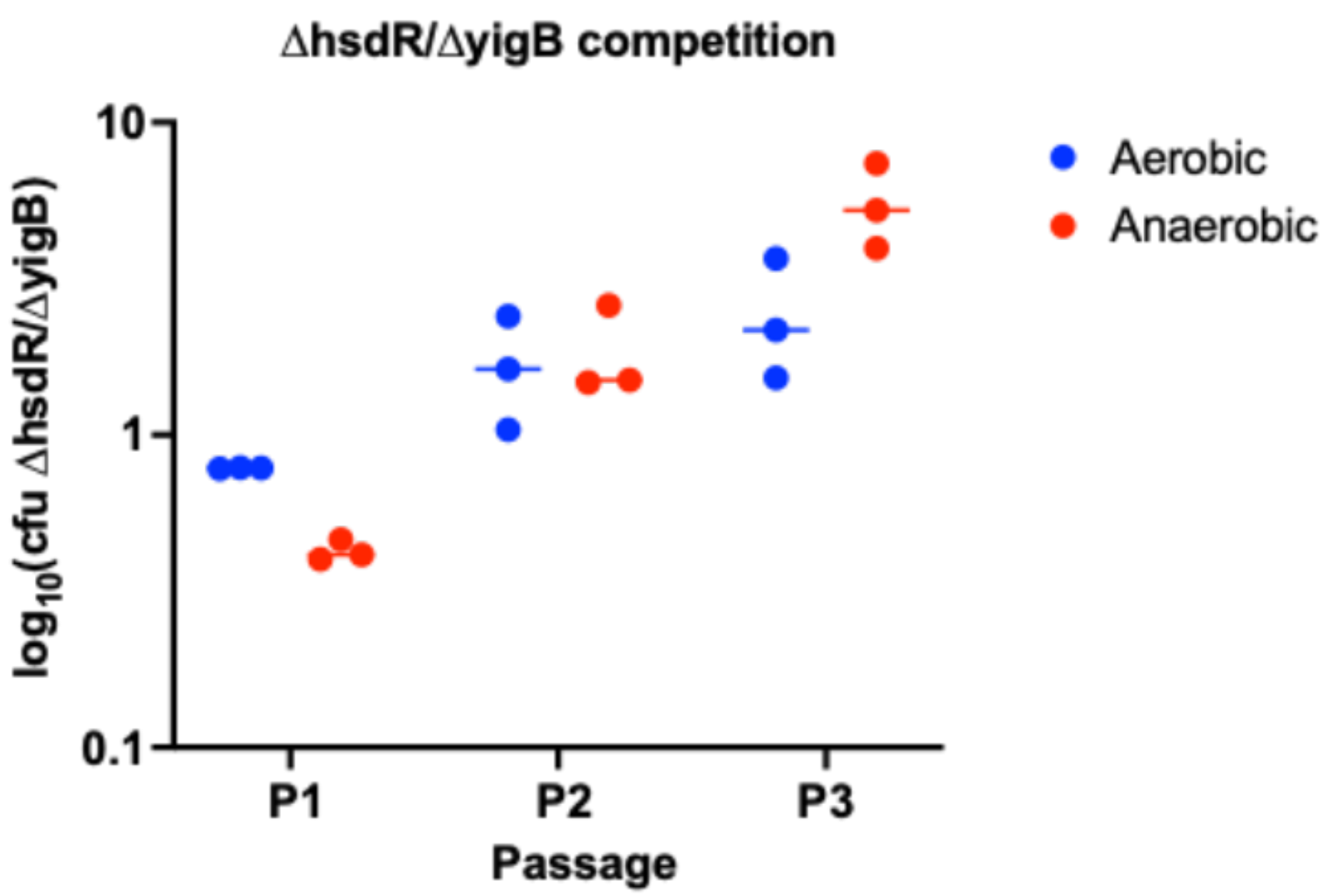

Figure S12. Competitive fitness of pigmented and non-pigmented strains under aerobic and anaerobic growth conditions. Pigmented (WT) and non-pigmented strain (ΔyigB) were mixed and serially passaged for three consecutive days under aerobic or anaerobic conditions. At each passage, the relative abundance of each strain was determined by selective plating and expressed as the log10 ratio of pigmented to non-pigmented CFU. Individual points represent biological replicates and horizontal lines indicate the mean. No aerobic specific competitive advantage of the pigmented strain was observed over the three passages.

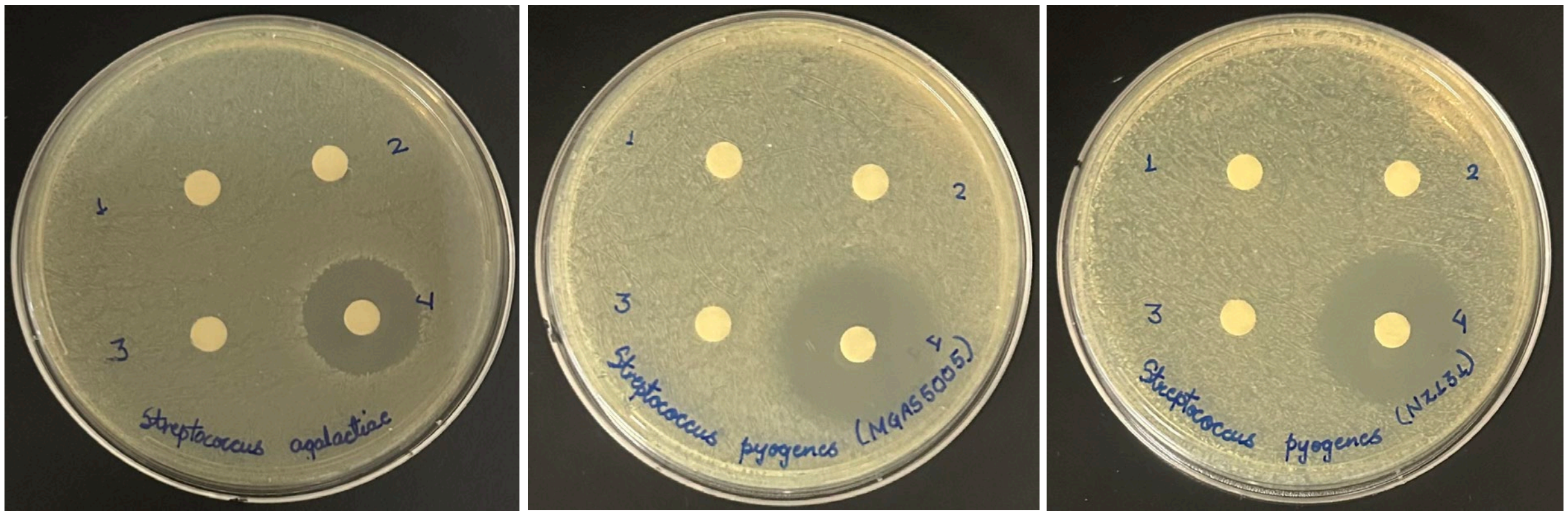

Streptococcus agalactiae A909 | *Streptococcus pyogenes* MGAS5005 | *Streptococcus pyogenes* *NZ131*

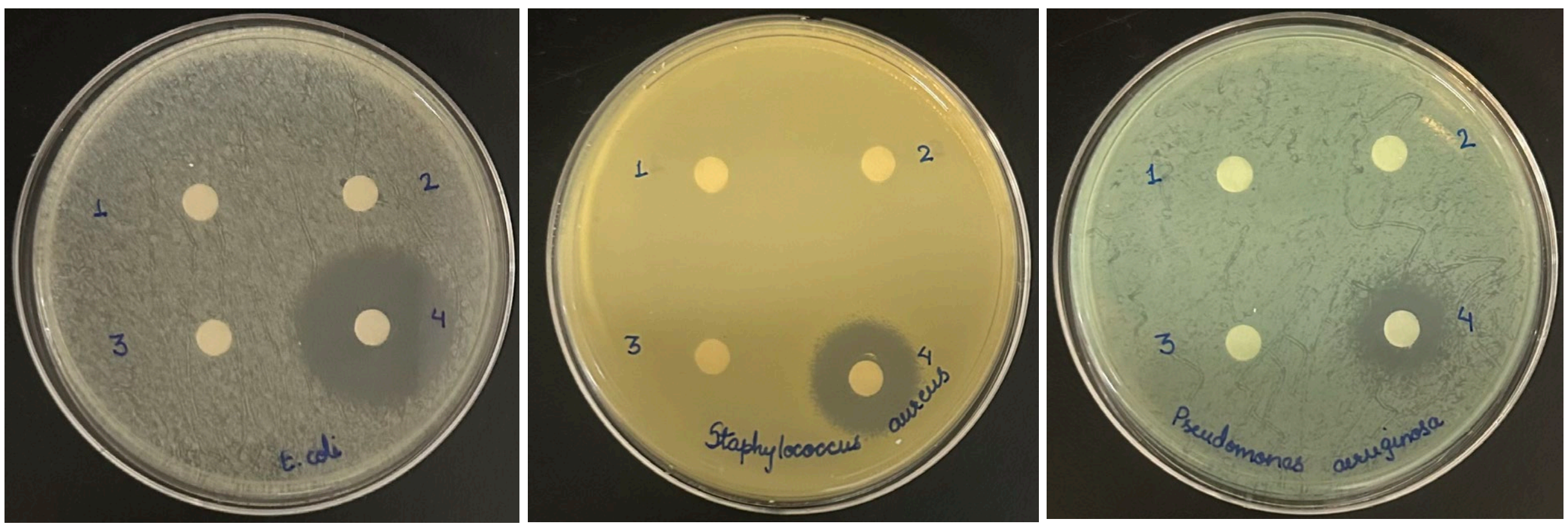

E. coli | *Staphylococcus aureus* NRS123 | *Pseudomonas aeruginosa* PAO1

Figure S13. Antibacterial test of pigmented and non-pigmented extracts. Disc diffusion assays were performed against *Streptococcus pyogenes*, *Staphylococcus aureus*, *Streptococcus agalactiae*, *Escherichia coli*, and *Pseudomonas aeruginosa*. THY agar was used for *S. pyogenes*, *S. aureus*, and *S. agalactiae*, while LB agar was used for *E. coli* and *P. aeruginosa*. Extracts of MGAS5005 (pigmented, Disc 1) and non-pigmented *purM::ISS1* (Disc 2); Discs were loaded with 10 µL of 1 mg/mL extracted material. Disc 3: PBS as a vehicle control; Disc 4: chloramphenicol (10 µL, 3 mg/mL) as a growth inhibitory control.

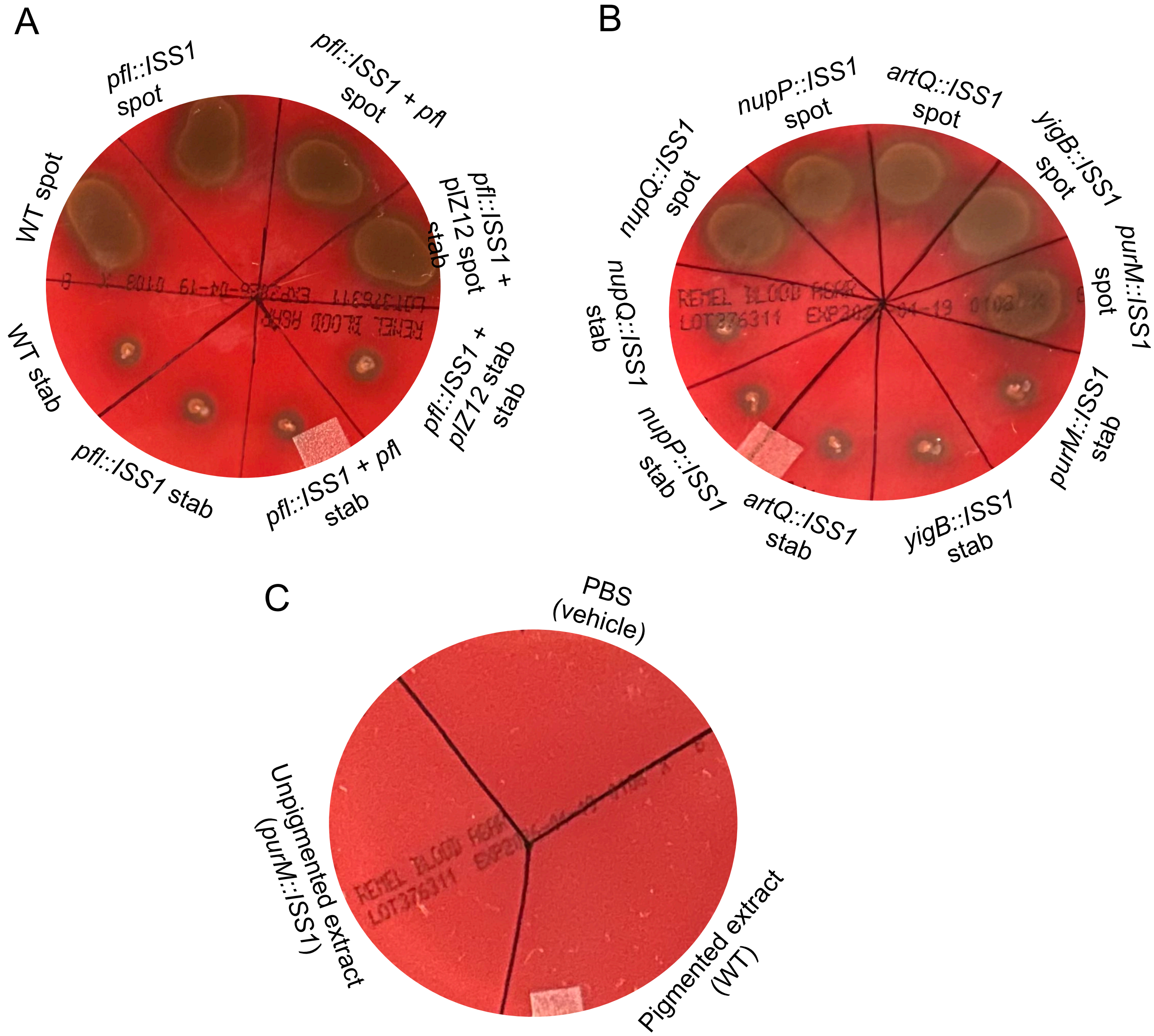

Figure S14. Hemolytic activity of pigmented and non-pigmented strains and their extracts. (A,B) Hemolytic activities of the MGAS5005 WT (pigmented) and non-pigmented mutant strains spotted onto or stabbed into blood agar showed no observable differences between WT and mutant strains. (C). Hemolytic activity assessed using 10 µL of pigmented and non-pigmented extracts (1 mg/mL) revealed no detectable activity for any of the extracts.

Table S1. Strains used in this study

Strains	Description	Ref
NV6	NV1 ΔhsdR::ery, Δcas9::Ptet-dcas9, tetR, tetM	Bjånes, Elisabet, et al. "An efficient in vivo-inducible CRISPR interference system for group A Streptococcus genetic analysis and pathogenesis studies." MBio 15.8 (2024): e00840-24.
MGAS5005	Wild-type M1 GAS	Hoe et al., 1999, Sumby et al., 2006 -> see Chang Mol Micro 2015 references
MGAS5005 Δrgg2		Chang, J.C., Jimenez, J.C. and Federle, M.J. 2015. Molecular Microbiology, 97: 1097-1113. https://doi.org/10.1111/mmi.13088
MGAS5005 Δrgg3		Chang, J.C., Jimenez, J.C. and Federle, M.J. 2015. Molecular Microbiology, 97: 1097-1113. https://doi.org/10.1111/mmi.13088
MGAS5005 Δrgg2 Δrgg3		Chang, J.C., Jimenez, J.C. and Federle, M.J. 2015. Molecular Microbiology, 97: 1097-1113. https://doi.org/10.1111/mmi.13088
MGAS5005 ΔpepO		Wilkening, R.V., Chang, J.C. and Federle, M.J. 2016 Molecular Microbiology, 99: 71-87. https://doi.org/10.1111/mmi.13216
MGAS5005 ΔhsdR::ery		This study
MGAS5005 ΔhsdR::ery ΔyigB::aphA3		This study
MGAS5005 ΔhsdR::ery Δfps::aphA3		This study
MGAS5005 ΔhsdR::ery ΔnupPQ::aphA3		This study
MGAS5005 ΔhsdR::ery Δpfl::aphA3		This study
MGAS5005 ΔhsdR::ery ΔatmDE::aphA3		This study
MGAS2221	Wild-type M1 GAS	Sumby, Paul, et al. "Evolutionary origin and emergence of a highly successful clone of serotype M1 group A Streptococcus involved multiple horizontal gene transfer events." The Journal of infectious diseases 192.5 (2005): 771-782.
MGAS5448	Wild-type M1 GAS	Kansal RG, Aziz RK, Kotb M., Modulation of expression of superantigens by human transferrin and lactoferrin: a novel mechanism in host-Streptococcus interactions, J Infect Dis, 2005, vol. 191 (pg. 2121-2129)
SF370	Wild-type M1 GAS	Ferretti, Joseph J., et al. "Complete genome sequence of an M1 strain of Streptococcus pyogenes." Proceedings of the National Academy of Sciences 98.8 (2001): 4658-4663.
NZ131	Wild-type M49 GAS	McShan, W. Michael, et al. "Genome sequence of a nephritogenic and highly transformable M49 strain of Streptococcus pyogenes." Journal of bacteriology 190.23 (2008): 7773-7785.
NZ131 Δrgg2		Chang, J.C., Jimenez, J.C. and Federle, M.J. 2015. Molecular Microbiology, 97: 1097-1113. https://doi.org/10.1111/mmi.13088
NZ131 Δrgg3::cat		Chang, J.C., Jimenez, J.C. and Federle, M.J. 2015. Molecular Microbiology, 97: 1097-1113. https://doi.org/10.1111/mmi.13088

Table S2. Plasmids and Primers used in this study.

Plasmid	Description	Ref
pGH9:ISS1	Temperature sensitive integration vector carrying ISS1 for insertional mutagenesis; ermR	Maguin, Emmanuelle, et al. "Efficient insertional mutagenesis in lactococci and other gram-positive bacteria." Journal of bacteriology 178.3 (1996): 931-935.
pJC159	Shuttle vector; temperature-sensitive; cmR	Chang, J.C., Jimenez, J.C. and Federle, M.J. 2015. Molecular Microbiology, 97: 1097-1113. https://doi.org/10.1111/mmi.13088
pJC229	Pshp3-lux reporter in pLZ12-Sp; spR	Chang, J.C., Jimenez, J.C. and Federle, M.J. 2015. Molecular Microbiology, 97: 1097-1113. https://doi.org/10.1111/mmi.13088
pJC496	pfl under its native promoter in pLZ12-Sp; spR	This study
pJC509	DhsdR::ery locus from NV6 cloned into pJC159; cmR	This study
pJC523	yigB upstream and downstream sequences flanking aphA3 in pJC159; cmR	This study
pJC525	S. pyogenes recA promoter cloned into pLZ12-Sp; spR	This study
pJC547	nupPQ cloned downstream of PrecA in pJC525; spR	This study
pJC548	atmDE cloned downstream of PrecA in pJC525; spR	This study
pJC549	fps cloned downstream of PrecA in pJC525; spR	This study
pLZ12-Sp	Shuttle vector; spR	Husmann LK, Scott JR, Lindahl G, Stenberg L (1995). Infect Immun 63: 345–348.
pTD01	yigB cloned downstream of PrecA in pJC525; spR	This study

Primers	
JC243	GGTTCATATTCATGTAGTGTTTAAGAACAAGAAGC
JC292	GCATGACGCGTATGGCTAAAATGAGAATATCACC
JC304	GCATGACGCGTCTAAAACAATTCATCCAGTAAAATATAA
JC320	CATGTTAATTAACGATACTATGTTATACGC
JC321	CATGTTAATTAAAGCGAACTTTTAGAAAAG
JC346	CATGGCGGCCGCGATTGGAATAGTCGGAATCG
JC347	CATGGCGGCCGCGTCTGTCCTACTTCCTTGC
JC348	CATGTTAATTAATATGGCGACAGTAATCTACG
JC349	CATGTTAATTAAGGTGATAGCTTGACTAGAAGG
JC623	TTCTAATAACATCACTTTCTCC
JC738	CATGCTGCAGCATTCTAATACAGTTTGTGACTG
JC739	CATGCTGCAGATAGCCATTTCATTTGTCTG
JC808	CATGGCGGCCGCCTGAGCTATCAATAGCATAAGG
JC809	CATGGTCGACCTCTGCCATGTGAGTCCTTTC
JC810	CATGGCGGCCGCCACCGCGGTGGGATCCTC
JC811	CATGGTCGACCTCGAGGGGGGGCCCG
JC816	CATGGCGGCCGCGGTCATTAAATGTATTGAAAACC
JC835	CATGGCGGCCGCATGTTATATTGCCACTTATCGG
JC836	CATGGTCGACTTTAGATAAAGGTAAGGACTTG
JC837	CATGACGCGTCATGAGTCTCTTTTCTTTTC
JC838	CATGACGCGTTAGATGAAACCATAATAGC
JC919	GTCCAACTAAGTGAAAGG
JC920	TTTTAGCCATTAATCATCCATATCTACTTCTG
JC921	TGGATGATTAATGGCTAAAATGAGAATATCAC
JC922	CTTTAGGCATCTAAAACAATTCATCCAGTAAAATATAATATTTTATTTTC
JC923	ATTGTTTTAGATGCCTAAAGAAAGAGTAG
JC924	TTTTAGCCATGACCTTCCTCCTTATGAATTG
JC925	GAGGAAGGTCATGGCTAAAATGAGAATATCAC
JC926	TAATATAACGCTAAAACAATTCATCCAGTAAAATATAATATTTTATTTTC
JC927	ATTGTTTTAGCGTTATATTAGTAAGCCTCTTAG
JC928	AAAGTCTATGGACAAGACG
JC929	TTTTAGCCATAAATTTCTCCTTAAATTCTAGTAACAAAGC
JC930	GGAGAAATTTATGGCTAAAATGAGAATATCAC
JC931	TTAGATACTACTAAAACAATTCATCCAGTAAAATATAATATTTTATTTTC
JC932	ATTGTTTTAGTAGTATCTAAATGATCAATAACTTATAAAAAAG
JC943	CATGGCGGCCGCATGTCTAAAAATGCGCAAAA
JC944	CATGAGATCTTTATTTAGATTTAATATAGTTGATTC
JC945	CATGGCGGCCGCATGAATGAAGCAATAATTCAA
JC946	CATGACGCGTTTATTTATGACTAAACCTTCTAG
JC947	CATGGCGGCCGCATGGACAAATTAGCTAGAAT
JC948	CATGACGCGTTTAGGCATTAAGTCGTAAC
JJ92	CATGGCGGCCGCTCTCCTCTTTTTTAACAAACC
JJ205	CATGGAATTCGTCCGTAAAACTTTATTAAAAC
SP001	CATGGTCGACCCCCTAACAGGCGGATACCAG
TD1	CATGGCGGCCGCATGATTACTGCTATTGTTTTTG
TD2	CATGAGATCTCTATCTGGCATATTTTAAGC

Table S3: Strain library assessed for pigment production.

Strain	M-type	Pigment
5448	1	**pink**
MGAS2221	1	**pink**
MGAS5005	1	**pink**
SF370	1	non-pigmented
02C1168	2	non-pigmented
GAS 188	3	non-pigmented
950771	3	non-pigmented
MGAS315	3	non-pigmented
48149	4	non-pigmented
GA19681	6	non-pigmented
JRS4	6	non-pigmented
MGAS10394	6	non-pigmented
37792	12	non-pigmented
HSC5	14	non-pigmented
MGAS8232	18	non-pigmented
BSPY033	28	non-pigmented
29487	33	non-pigmented
BSPY122	33	non-pigmented
SS795	41	non-pigmented
CS101	49	non-pigmented
NZ131	49	non-pigmented
Alab49 WT	53	non-pigmented
59217	77	non-pigmented
BSPY047	78	non-pigmented
CK409	78	non-pigmented
CT95-157	78	non-pigmented
D812	78	non-pigmented
58362	89	**pink**
02C1147	89	non-pigmented
02C1163	89	non-pigmented
BSPY049	89	**yellow**
MGAS2017	89	non-pigmented
TW159	89	non-pigmented
C506	91	non-pigmented
A756	96	**very yellow**
BSPY076	101	**fairly yellow**
D641	101	non-pigmented
TW128	103	non-pigmented
NS030	108	non-pigmented
3018-01	114	non-pigmented
SS1681	St98054	**yellow**
